# Challenges of community participation in health emergency and disaster risk management in an Iranian context: a qualitative study

**DOI:** 10.3389/fpubh.2025.1460421

**Published:** 2025-01-28

**Authors:** Mohammad Azim Mahmodi, Mehrdad Farrokhi, Seyyed Mohammad Reza Hosseini, Mehdi Najafi, Mohammad Esmaeel Motlagh, Hamid Reza Khankeh

**Affiliations:** ^1^Health in Emergency and Disaster Research Center, Social Health Research Institute, University of Social Welfare and Rehabilitation Sciences, Tehran, Iran; ^2^Department of Health in Emergencies and Disasters, School of Nursing and Midwifery, Birjand University of Medical Sciences, Birjand, Iran; ^3^Department of Rescue and Relief, Iran Helal Applied Science Higher Education Institute, Tehran, Iran; ^4^School of Medicine, Jundishapur University of Medical Sciences, Ahvaz, Iran

**Keywords:** community participation, risk management, social capital, perception, health, disasters, emergencies

## Abstract

**Background:**

Disasters are considered one of the major threats to the health of communities. Given the global spread of disasters, there has been a renewed emphasis in recent years on using community participation approaches in disaster risk management. Community participation in field of emergency and disaster health in Low and Middle-income countries faces a lot of challenges.

**Objective:**

The objective of this study is to explore the specific challenges faced by communities in Iran concerning their participation in health emergency and disaster risk management.

**Methods:**

In this study, a qualitative content analysis as research methodology was employed. Purposeful sampling was conducted from June 2023 to May 2024 among community members who were willing to participate and had experience collaborating during disasters. Data were collected through semi-structured in-depth interviews and analyzed using Graneheim and Lundman’s method. The rigor of the study was ensured using Lincoln and Guba’s criteria. Each interview was recorded, transcribed into a word document, and then uploaded into MAXQDA (2020). A total of 23 interviews were conducted with 20 participants.

**Results:**

There were 15 males and five females participating in the study including healthcare managers, academic people, and laypeople. Data analysis revealed nine distinct categories, which were grouped into three overarching themes based on their similarities. These themes addressed issues of insufficient risk perception, community traumatization, and poor social capital.

**Conclusion:**

This study sheds light on the multifaceted challenges that hinder effective community participation in health emergency and disaster risk management in Iran. Addressing these barriers is essential for enhancing community resilience and ensuring effective disaster preparedness. To overcome these challenges, it is imperative for policymakers, healthcare managers, and community leaders to collaborate and implement comprehensive strategies that foster community participation.

## 1 Introduction

Disasters caused by natural hazards, such as earthquakes and floods, as well as those stemming from man-made hazards, like chemical spills and acts of terrorism are considered one of the major threats to the health of communities (1). According to the latest EM-DAT report, 2023 saw 399 natural hazard-related disasters worldwide, affecting 93.1 million people and resulting in 86,473 deaths (2). Iran, as a disaster-prone country, has experienced numerous disasters over the past century (3). Over 80,000 Iranians lost their lives as a result of disasters, including earthquakes and floods, between 1990 and 2020 (4, 5). In the upcoming years, it is expected that more events linked to climate change, like dust storms and floods, will occur, exacerbating the risks to community health (6).

In the past two decades, the emphasis on disaster risk management (DRM) has surged as disasters grow in frequency and severity (7, 8). However, constrained resources have led to a shift from government-centered models to community-based approaches, as emphasized in the Sendai Framework for Disaster Risk Reduction 2015–2030. This framework promotes inclusive, people-centered strategies, encouraging communities to play an active role in preparedness and resilience building (9, 10).

Community participation (CP) enhances DRM by building local capacities, improving public awareness, fostering trust between the public and the government, and better aligning programs with local conditions (11). According to the World Health Organization guideline and the study by Brunton et al. (13), a spectrum of CP have been defined, which include informing, consulting, involving, collaborating, and empowering (12, 13). It is noteworthy that all these approaches are crucial for improving the health status of vulnerable populations.

The principal religious scripture of Islam, the Holy Quran, provides guidance on various aspects of life, including health and well-being. Numerous verses highlight the significance of maintaining good health and taking preventive actions to avoid harm (14). As an example, Quran 2:195 states: “And make not your own hands contribute to your own demise, for the Lord loves the actions of good.” This verse emphasizes the necessity of taking precautionary measures to safeguard against harm and promote well-being. By adopting preventive strategies and encouraging CP, individuals and communities can reduce their vulnerability to disasters and strengthen their resilience (15).

Communities, with their deep understanding of local realities, are uniquely positioned to identify both opportunities and constraints, enabling them to play a crucial role in reducing health risks associated with disasters through preventive actions and fostering resilience (16). Also by involving community members directly in disaster preparedness education, establishing responsive communication and early warning systems, and offering psychological and social support, communities empower themselves to manage risks. Local volunteers and collective engagement in disaster planning enable rapid, efficient responses tailored to local needs (17, 18). Furthermore, when individuals and families take steps to prepare, it strengthens the community’s collective capacity to prevent health impacts such as preventable deaths, injuries, infectious diseases outbreak, and mental health issues (19–21).

Despite CP’s recognized importance in health emergency and disaster risk management (EDRM), numerous obstacles, including managerial, socio-cultural, and policy challenges, impede its full implementation, particularly in low- and middle-income countries (LMICs) (22). In Iran—a middle-income country frequently struck by disasters—research on CP’s Challenges in health EDRM remains limited. This qualitative study addresses this gap by exploring the experiences of community members, providing insights to inform policymakers and health EDRM stakeholders. Alongside contributing to the body of knowledge, the findings offer practical recommendations to enhance CP in health EDRM, not only benefiting Iran but also informing similar settings globally.

## 2 Materials and methods

### 2.1 Research paradigms

To explore the challenges of CP in health EDRM in Iran, a qualitative content analysis was conducted from June 2023 to May 2024. This approach was chosen for its effectiveness in uncovering nuanced insights, providing a thorough understanding of participants’ perspectives, beliefs, and experiences related to the subject. Through this method, researchers could interpret and uncover deeper meanings within the text, thus enhancing knowledge in the field and effectively addressing the research questions (23, 24).

### 2.2 Context

Iran, a country with a population exceeding 83 million, is situated in Western Asia. It is considered highly vulnerable due to its geographical exposure to natural disasters, socio-economic challenges, and structural inequalities. Positioned in a seismically active region, Iran frequently experiences earthquakes, floods, and droughts, which heighten the communities’ exposure to severe natural hazards. Additionally, economic difficulties and social inequality exacerbate this vulnerability by limiting access to resources, preparedness measures, and resilience-building opportunities for large portions of the population (4). The Global Risk Report identifies Iran as one of the Middle East’s most vulnerable countries and ranks it among the world’s highly vulnerable countries. The report emphasizes that regions with both environmental and economic vulnerabilities face amplified risks, as resource constraints hinder effective disaster management and recovery capabilities (25). Iran’s population is also ethnically diverse, with Persians making up 54%, followed by Azerbaijanis (16%), Kurds (10%), Gilaks (7%), Lurs (6%), and smaller percentages of Turkmen (2%), Arabs (2%), and Balochs (2%) (26).

### 2.3 Study participants

The study’s participants comprised healthcare managers, academic people, and laypeople who had experience collaborating during disasters. Those who agreed to participate were selected through purposive sampling, with a maximum variety regarding their personal and position experience. Healthcare managers were individuals working in the hospital and emergency medical system affiliated with the Ministry of Health such as head nurses, supervisors, and matrons. The academic people mostly consisted of faculty members and doctoral students specializing in emergency and disaster health. Laypeople were those who volunteered in organizations affiliated with the Red Crescent. Interviews were conducted with healthcare managers at their workplace, academics at universities, and laypeople at their residence or workplace. The study’s inclusion criteria were having collaborating experience in at least two disasters, having a minimum of 2 years of managerial experience, having the ability to communicate effectively, and agreeing to participate in the study. The exclusion criteria included an unwillingness to continue to participate in the interview and a lack of trust in disclosing personal experiences.

### 2.4 Data collection

The first author, a PhD candidate, conducted in-depth, semi-structured interviews in a quiet, private setting to gather data. Individual introduction meetings preceded the interviews to brief participants on the study’s purpose and get their permission to record the interviews. After that, each participant’s written agreement was obtained, and the interview process was discussed. Furthermore, the participants were consulted regarding the place and schedule of the interview. Demographic data, including age, gender, experience managing health care after disasters, and educational background, was collected before the interview ever began. The interviews then proceeded with initial, middle, and final questions. “Please share your experience from the last disaster you were involved in” was an example of an initial question. “What role do you see for yourself and others in CP in health EDRM?” and “What is your experience with the current challenges impacting CP in health risk management before disasters strike?” are two examples of middle questions aimed to delve deeper into participants’ experiences. Probing questions like “Please elaborate on this” and “What do you mean?” were also utilized. The final question invited participants to share anything they might have missed during the interview. When category saturation was achieved and no new information emerged, data collection was terminated. Interviews were conducted with 20 participants who had experience of participation in health EDRM, including six healthcare managers, seven academic people, and seven laypeople. In total, 23 interviews were conducted (20 initial and 3 follow-up). The follow-up interviews aimed to clarify ambiguities from the initial sessions with three participants. The initial in-depth interviews lasted between 45 and 60 min, averaging 50 min, while the follow-up interviews lasted between 10 and 20 min, averaging 15 min.

### 2.5 Data analysis

In qualitative research, data analysis starts and unfolds alongside data collection (27). This study employed the widely accepted five-step method of qualitative content analysis developed by Graneheim and Lundman (24, 28). Initially, the recorded interviews were listened to multiple times and transcribed meticulously. In the next phase, prior to conducting any analysis, the texts were reviewed several times to grasp the insights and perspectives shared by the participants. Following this, each interview was evaluated as a complete entity, with the essential meaning or main ideas articulated in one or more sentences. In the subsequent step, subcategories were derived from the codes, categories were formed based on the subcategories, and overarching themes were developed from the categories. To ensure the accuracy and reliability of the findings, copies and initial codes were reviewed by several participants (member checking). Additionally, to validate the study, two individuals performed the coding of the interviews, and ultimately, a disaster specialist refined, combined, or removed codes and categories as necessary. The coding method was first aided using MAXQDA (2020) software, but later phases of qualitative content analysis were done manually.

### 2.6 Trustworthiness

To ensure the trustworthiness of the study, criteria outlined by Lincoln and Guba were considered, such as credibility, dependability, confirmability, transferability, and authenticity (29). In terms of credibility, prolonged engagement (10 months), ongoing observation of the subject matter, effective communication with participants, colleagues’ peer reviews, member checking with participants, and deep immersion in the data were employed in order to satisfy this. To ensure the dependability of the data from the outset of the study, a focused review of the literature was conducted to minimize potential researcher bias in data collection and analysis. Confirmability was achieved in two ways: through an external peer review and the inclusion of direct quotations that reflected participants’ actual experiences. During this process of external review, two experienced qualitative researchers assessed and validated the accuracy of the data analysis. To improve the applicability of the findings (transferability), detailed descriptions of participant backgrounds and the study context were provided, enabling readers to assess the relevance of the findings to their own circumstances. The researcher upheld ethical standards by obtaining informed consent from participants, respecting their statements, fostering rapport, and ensuring clarity about the research methodology, thereby maintaining authenticity throughout the study.

### 2.7 Ethical considerations

This study is part of a doctoral thesis in emergencies and disasters health, registered under the ethics code IR.USWR.REC.1402.086. It adheres to ethical guidelines for human subjects, ensuring the confidentiality of information, voluntary participation, and the right for participants to withdraw at any time.

## 3 Results

The majority of participants in this study were male (*n* = 15; 75%), and the majority had a master’s degree (*n* = 11; 55%). The participants’ mean age was 42.6 years. Furthermore, six individuals (30%) were health care providers, possessing an average of 19 years of managerial experience. The participants’ average number of disaster experiences was approximately 5.8 (Table 1).

**Table 1:** Demographic information of the participants.

Participants	Gender	age	Disaster experience (number)	Management experience (years)	Degree of education
P_1_	M	47	9	21	MSc
P_2_	M	43	9	–	PhD
P_3_	F	40	2	9	MSc
P_4_	M	53	8		MSc
P_5_	F	42	10	–	PhD
P_6_	M	39	3	–	MSc
P_7_	M	40	5	–	BSc
P_8_	M	46	8	26	PhD
P_9_	F	39	4		BSc
P_10_	M	37	6	–	PhD candidate
P_11_	M	43	9	–	PhD
P_12_	M	40	2	14	PhD
P_13_	M	55	8	27	MSc
P_14_	F	42	6	–	MSc
P_15_	M	52	4	–	BSc
P_16_	M	34	5	–	PhD candidate
P_17_	M	46	2	17	PhD
P_18_	F	41	3		MSc
P_19_	M	33	7	–	PhD candidate
P_20_	M	40	6	–	PhD

Following data analysis, 672 open codes, 35 subcategories, nine categories, and three themes were identified. The three main themes, representing the challenges related to CP in health EDRM, included insufficient risk perception (RP), community traumatization, and poor social capital (Table 2).

**Table 2:** Subcategories, categories and themes obtained from analysis of interviews.

Theme	Category	Subcategory
Insufficient risk perception	Community characteristics diversity	Individual attributes (aging, low educational level)
		Social attributes (poverty, inequality)
		Cultural attributes (religious beliefs, languages, dialects)
	Inadequate community risk assessment	Insufficient specialized knowledge in identifying risks
		Insufficient availability of reliable and accurate information sources
		The deficiency of necessary tools and methods
		The insubstantial practical experience in RA
	Poor risk communication	The inadequacy of alert systems
		Failure to transmit accurate and precise information
		Poor health-related media campaigns
		Weakness of communication channels
Community traumatization	Increased feeling of unsafety in community	Recurrent experiences of disasters
		Negative experiences of previous inefficient responses
		Lack of effective health DRM plan
		Inadequate performance of media during disasters
	Inadequate psychological preparedness	Economic instability
		Inadequacy of social support
		Emotional suppression
		Limited stress coping skills
	Reliance on fatalistic attitude	Lack of effective health-related social structures
		Deficiency of problem-solving skills education
		Different interpretations of religious teachings
		Belief in the inability to change conditions
Poor social capital	Ineffective supportive social networks	Class disparities
		Ethnic diversity
		Weak community interactions
		Scarcity of resources
	Weakened social norms	Lack of cooperative spirit
		Disregard for social cohesion
		Weakened ethical beliefs
		Reduced in a sense of responsibility
	The erosion of mutual trust	Top-down structures
		Lack of transparency of healthcare managers
		Poor foresight healthcare managers
		Insufficient attention to the local community’s needs

### 3.1 Insufficient risk perception

This theme suggests that some community issues, such as community characteristics diversity, inadequate community risk assessment (RA), and poor risk communication (RC), can significantly influence CP in health EDRM.

#### 3.1.1 Community characteristics diversity

This category highlighted that individual attributes (such as aging and low educational level), social attributes (such as poverty and inequality), and cultural attributes (such as religious beliefs, languages, and dialects) within communities pose significant challenges to CP in health EDRM. A PhD candidate in disaster and emergency health (P5) noted:

*“During the Kermanshah earthquake, I was on-site and realized that the use of local languages and dialects in the community led to communication issues, impacting the efficiency and effectiveness of DRM programs. Most individuals in that community could not properly receive vital information and were unable to actively participate in collaborative processes to mitigate health risks.”*

Regarding cultural attributes, (P1) mentioned this:

*“From my perspective, religious diversity in communities can lead to differing opinions and approaches to health issues. Some communities, due to their specific religious beliefs, may be hesitant to accept or implement health recommendations and preventive measures. For example, during the COVID-19 pandemic, particularly when vaccination was recommended, I observed that a segment of the Iranian community did not participate as required, which in turn complicated health outcomes.”*

Additionally, the aging population and the lack of sufficient education on health risks related to disasters can significantly hinder CP in this regard. As P7 pointed out, the increasing number of older adults in communities is a significant concern:

*“Our communities are moving towards aging. Older adults populations have specific needs and greater vulnerabilities. I have come to understand that if seniors receive recommendations tailored to their physical and cognitive conditions, they can participate more effectively compared to others. Unfortunately, there is less focus on different age groups in our communities, and even education is often provided in a generalized manner.”*

P12, with his experience in disaster management, highlighted the scarcity of health risk education related to disasters within the communities:

*“While organizations have diverse educational programs, I have not yet encountered effective community-wide education aimed at increasing public awareness of preventive methods and health risk mitigation before disasters occur. I believe this education gap could lead to reduced CP and ultimately exacerbate health outcomes.”*

#### 3.1.2 Inadequate community RA

The lack of specialized knowledge in identifying and assessing risks, the insufficient availability of reliable and accurate information sources for RA, the deficiency of necessary tools and methods, and the insubstantial practical experience in RA within the communities are factors that can impact the communities’ performance in identifying their health risks. These factors lead to an inadequate perception of health consequences and ultimately result in reduced CP. P17, referring to himself as laypeople, stated:

*“In my opinion, many individuals in our community, like myself, lack sufficient knowledge to identify health risks arising from disasters for themselves and their families. This ignorance among me and similar individuals prevents us from participating in preventive measures and ultimately can lead to our community’s unpreparedness and inability to reduce health risks.”*

P19 mentioned in this context:

*“To conduct a RA for an organization, I required a hazard map for that specific area of the city. However, such a map was not available. Additionally, there is no single tool or method compatible with our communities that ordinary people can use. In my opinion, there is still a lack of reliable and accurate information resources and tools necessary for identifying and analyzing health-related risks in our communities, which can pose a fundamental challenge to CP.”*

P2 commented on the development of CP in reducing health risks:

*“In our high-risk community, where we face numerous events, it would be beneficial to have practical experience in the RA process for identifying health impacts from disasters, even if just once. I think that lacking experience and familiarity with this process can keep us vulnerable to risks and result in weaker CP.”*

#### 3.1.3 Poor RC

This category draws attention to the inadequate community-level RC that might impede appropriate RP and result in lower CP in health risk reduction initiatives. The inadequacy of alert systems, the failure to transmit accurate and precise information, poor health-related media campaigns, and the weakness of communication channels play a significant role in the deficiency of RC.

The inadequacy of alert systems emerged as a key challenge hindering CP, as clearly evident in the findings of this research. A faculty member in disaster and emergency health (P20) remarked:

*“I believe that if an integrated communication system existed within communities, we could witness the accurate dissemination of critical information, particularly regarding health risks, even before disasters strike. This would not only facilitate information exchange but also enhance coordination among* var*ious agencies. Unfortunately, this shortcoming persists in our communities, leading to an increase in the challenges associated with pre-disaster health risk management.”*

Inaccurate and unclear information dissemination poses a significant challenge in the realm of RC. According to the experience of one participant, who stated (P13):

*“The sending of inappropriate and vague warning messages regarding weather-related dangers is a common experience among the public. This leads to confusion and a lack of coordination among community members and relevant agencies, ultimately resulting in increased casualties and losses. This is because individuals lack awareness of the appropriate actions and responses to take in such situations.”*

Another identified issue in this qualitative research was poor health-related media campaigns in the health sector. This hinders establishing effective communication with the community and sharing reliable information about health issues. One of the study participants (P17) expressed this matter as follows:

*“With years of experience working in the healthcare system, I must admit that we face a lack of effective and specialized media in this field. This gap has so far prevented establishing proper and reliable communication with the community regarding health. In my opinion, this problem has not only negatively impacted the level of awareness and preparedness in the community but has also made the active participation of the community in managing health-related risks challenging.”*

The weakness of effective communication channels hinders the timely delivery of vital information to the community and prevents necessary coordination between institutions and the community. One participant in the study (P13) clearly articulates this issue:

*“During disasters like COVID-19 and floods, where rapid dissemination of information to all members of the community is crucial, I have repeatedly witnessed the community’s lack of access to essential news and information. This occurs due to the inadequacy of communication channels. This issue prevents the timely delivery of vital information to community members and the establishment of necessary coordination between institutions and the community. Ultimately, this leads to poor decision-making and increased harm.”*

### 3.2 Community traumatization

This theme suggests that a range of factors, including increased feeling of unsafety in community, inadequate psychological preparedness, and reliance on a fatalistic attitude, can lead to traumatization of the community at different levels, affecting individuals, families, social groups, and societal institutions. As a result, community traumatization may have a major effect on CP in health EDRM.

#### 3.2.1 Increased feeling of unsafety in community

Feelings of unsafety within a community hinder individuals from participating in long-term, sustainable activities. In such conditions, individuals often prioritize short-term concerns that offer immediate security rather than preparing for future disasters. Based on findings, these feelings stem from recurrent experiences of disasters, negative experiences of previous inefficient responses, the lack of effective health EDRM plan, and the inadequate performance of media during disasters. One study participant (P15) emphasized the harmful effects of repeated disaster experiences on CP.

*“In a community that has repeatedly experienced disasters, these experiences leave deep wounds in the fabric of the community. Each time these wounds reopen, they evoke painful memories of the past, leading to chronic fear and anxiety. This erodes hope for the future and diminishes the community’s ability to respond timely to health concerns.”*

Witnessing the inefficacy of disaster response can leave a lasting negative impact on the community. This experience can lead to the belief that nothing can be done to prevent disasters or mitigate their effects. Consequently, individuals lose motivation to participate in health EDRM activities. Another participant (P9) expressed a disillusioned tone regarding the disregard of opinions and suggestions from affected communities in decision-making and actions related to disasters:

*“When those of us directly affected by disasters witness our opinions and suggestions being ignored in decisions and actions related to disasters, what motivation or hope remains for us to participate in this field?”*

The lack of effective health EDRM plan to deal with disasters can foster a sense of unsafety within communities. One participant (P14) expressed this issue as follows:

*“We are constantly witnessing recurring events like traffic accidents that have irreparable health consequences every year. However, not only are there no plans formulated to prevent these incidents year after year, but the existing plans also fail to evolve. This lack of transparent and comprehensive planning ultimately leads to confusion and a sense of unsafety among community members.”*

During disasters, the dissemination of inaccurate, misleading, or biased information by media outlets can intensify fear, anxiety, and confusion within affected communities. Consequently, this can undermine CP in preventive measures and health risk management. As one participant (P10) noted:

*“When incorrect or misleading information is circulated within a community, it instills fear and anxiety, impairing their ability to make informed decisions and take effective actions. Furthermore, the lack of timely and accurate news coverage can intensify these fears, anxieties, and confusions.”*

#### 3.2.2 Inadequate psychological preparedness

A community that is not psychologically equipped to cope with disasters will not be able to take the necessary steps to mitigate health-related risks before the event occurs. Factors such as economic instability, inadequacy of social support, emotional suppression, and the limited stress-coping skills play a role in this regard. One of the participants (P2) stated:

*“Economic problems affect many individuals and families in today’s communities. In my opinion, this uncertainty about the economic future can have negative consequences for both individuals and the community as a whole. For instance, communities might not have sufficient capacity to construct disaster-resistant buildings or insure their properties. Additionally, the stress arising from this situation can diminish their ability to cope with disasters.”*

Another participant (P6) stated:

*“The lack of proper support from family, friends, or community can make individuals feel isolated and lonely. This accordingly implies that in disaster situation, such people experience difficulties in accessing shelter as well as food and medications.”*

Moreover, the participant P15 stated:

*“In today’s communities, the expression of emotions, particularly negative ones such as anxiety, fear, and sadness, is being marginalized. The suppression of these emotions can lead to increased stress, anxiety, and mental health issues in individuals. Furthermore, this can hinder communication and seeking help from others.”*

Participant P11 also stated:

*“In my view, stress is a widespread phenomenon among individuals even at early ages in today’s communities. However, a less-discussed aspect is the shortcomings in societal behavior towards this issue. We lack the knowledge of how to effectively address this challenge, and this can hinder the community’s ability for rational thinking, sound decision-making, and effective actions to mitigate health risks.”*

#### 3.2.3 Reliance on fatalistic attitude

This belief, which is deeply embedded in the convictions of contemporary communities, can lead to passivity and a lack of willingness to actively participate in managing health risks before disasters occur. According to research findings, factors such as the lack of effective health-related social structures, deficiency of problem-solving skills education, different interpretations of religious teachings, and the belief in the inability to change conditions contribute to the tendency toward fatalism.

Strong and accountable civil institutions related to health, such as active associations, non-governmental organizations (NGOs), and local councils, can provide a platform for CP in decision-making and collective actions related to community health. The absence of these structures in contemporary communities deprives people of engaging in public affairs and marginalizes them. This weakens the sense of responsibility toward community health and hinders active CP in preventive measures and disaster preparedness. In the present study, one of the participants (P18) stated:

*“In our community, the lack of effective channels for voicing public concerns has fostered a sense of despair and isolation among community members. This situation entraps individuals within complex and inaccessible bureaucracies, reinforcing the belief that their efforts are futile and leading to apathy towards the future.”*

On the other hand, the lack of problem-solving skills training, such as critical thinking, creativity, and teamwork, deprives individuals of the necessary tools to face challenges and find innovative solutions. This reinforces the belief in a predetermined and unchangeable fate, as if placing invisible chains on their efforts and initiatives. Participant (P8) stated regarding this matter:

*“In communities deprived of education in creativity, innovation, and problem-solving skills, expecting leadership in addressing issues and advancing community affairs is a vain hope. This deficiency traps individuals in the belief of a predetermined fate and prevents their empowerment and active participation in tackling societal challenges, including pre-disaster health risk management.”*

In religious communities like ours, diverse interpretations of religious teachings can contribute to the formation or intensification of fatalistic beliefs. Participant (P5) noted:

*“In certain communities, natural disasters are perceived as divine punishment inflicted due to people’s sins. This belief can lead people to rely on prayer and supplication to God instead of making efforts to prevent disasters or prepare to cope with them.”*

The belief in the inability to change circumstances can also pave the way for the formation or intensification of fatalistic beliefs among individuals within a community. One of the participants (P9) stated regarding this:

*“During disasters, I have witnessed countless individuals within the community relying solely on governmental and relief services rather than their capabilities. This stems from a lack of belief in individual and collective abilities, hindering proactive initiatives and active participation aimed at improving living conditions. As long as individuals do not believe they can play a significant role in mitigating disasters and associated health risks, they will lack the motivation and necessary will to participate in preventive actions and preparedness efforts.”*

### 3.3 Poor social capital

Based on the participants’ experiences in this study, this theme suggests that deficiencies in relationships, norms, and prevailing values within the community can be significant barriers to active CP in health EDRM. Within communities, ineffective supportive social networks, weakened social norms, and the erosion of mutual trust all contribute to the depletion of social capital.

#### 3.3.1 Ineffective supportive social networks

This concept stems from a confluence of factors, including class disparities, ethnic diversity, weak community interactions, and a scarcity of resources necessary for active participation. Participant (P16), while referring to class disparities as a significant barrier to CP in health EDRM, stated:

*“I think that in the community we live in, unequal access to resources, opportunities, and information has reinforced a sense of inequality among different members of the community. Even during disasters, I have repeatedly witnessed that those who are financially stronger benefit more from available facilities. This issue leads to feelings of isolation among individuals in lower social classes and hinders their participation and cooperation.”*

Ethnic diversity primarily refers to the differences in ethnicity and race within a community. In communities with high ethnic diversity, strong social networks may not develop. Participant (P14) stated:

*“In my opinion, our country is very rich in ethnic diversity. In some major cities, like Tehran, one can observe almost every ethnicity. This diversity might contribute to a sense of distance among individuals in the community, especially in large urban areas.”*

The lack of close and meaningful relationships among community members can lead to isolation and a lack of sense of belonging to the community. This issue can serve as another barrier to CP in health EDRM. As Participant P12 aptly stated:

*“During the COVID-19 pandemic, we witnessed a decline in close relationships among individuals, even within families, which often led to mental health issues among individuals. For extended periods, families were unable to gather in person. Even now that some time has passed since then, the effects of this decline in relationships can still be observed.”*

The lack of essential resources, such as time and money, is a barrier to CP. Participant (P11) stated:

*“Currently, in our community, we need resources to participate in each other’s affairs. A few years ago, in a community, a flood warning was issued, but we had no means available to implement pre-flood mitigation measures.”*

#### 3.3.2 Weakened social norms

This category highlights that when norms and common values within a community weaken, CP in health EDRM is affected. Factors contributing to this include a lack of a cooperative spirit, disregard for social cohesion, weakened ethical beliefs, and reduced sense of responsibility. Participant (P9) remarked:

*“Today, we observe that in* var*ious communities, many individuals have a weak spirit of cooperation. Sometimes, they are indifferent to each other’s problems and do not help each other in times of need.”*

Another primary root of the community’s weak participation in health EDRM is social cohesion negligence. Participant (P4) stated:

*“In my view, nowadays individuals within our community have become indifferent to each other. Even families lack the necessary cohesion compared to the past, and this lack of cohesion spreads to the community itself.”*

Weak moral beliefs undermine the foundation of social order and jeopardize CP. Participant (P3) noted:

*“I think when individuals become indifferent to ethical principles such as honesty, justice, and compassion, the motivation to adhere to laws and fulfill social commitments diminishes. This issue makes participation in collective activities more challenging.”*

The decline in the sense of responsibility is also a major issue for communities regarding CP. One participant (P16) stated:

*“In my opinion, members of our community are currently neglecting their duties and commitments toward the community. They often use the proverb ‘No one will be buried in someone else’s grave,’ which reduces the motivation for active participation in addressing common problems.”*

#### 3.3.3 The erosion of mutual trust

This category demonstrates that a decline in societal trust toward each other, institutions, and their leaders negatively impacts their participation. Factors such as top-down structures, a lack of transparency among healthcare managers, poor foresight by these managers, and insufficient attention to the local community’s needs play a significant role in diminishing societal trust.

Top-down structures promote a culture of distrust within communities. When individuals feel they have no role in decision-making processes that affect their lives, they are less likely to trust institutions and their leaders. Participant P19 stated:

*“In my opinion, in our country, decision-making at both macro and even micro levels is concentrated in the hands of a limited number of individuals at the top of the hierarchy. This concentration leads to feelings of powerlessness and alienation among individuals at lower levels of the hierarchy, especially ordinary members of the community.”*

The lack of transparency among healthcare managers within the community is another barrier to CP. The participant (P20) stated:

*“I think when members of the community do not receive accurate and timely information about health risks and disaster preparedness plans from healthcare managers, especially through the media, it undermines their trust in them.”*

The inability to predict future events and the lack of necessary preparedness among healthcare managers today pose another challenge to CP. Participant P15 expressed:

*“In my opinion, when healthcare managers became aware of the spread of the COVID-19 virus, they did not take any action to prepare for this deadly disease until some individuals became infected, which resulted in distrust within the community.”*

Neglecting the specific needs and concerns of a particular community is another significant challenge for CP. Participant P11 stated:

*“In my view, when managers act in a community without considering local needs, this action will result in nothing but further distrust. We have often witnessed in post-disaster reconstruction processes that services provided without attention to the community’s needs have ended up being ineffective.”*

## 4 Discussion

This study aimed to explore the specific challenges faced by communities regarding CP in health EDRM. The study’s findings reveal three prominent themes: inadequate RP, community traumatization, and poor social capital. These themes shed light on critical challenges that affect the community’s ability to effectively manage health-related risks. The summary of the comparative analysis of our study and previous research is presented in Table 3.

**Table 3:** Summary of comparative analysis of CP’s challenges in Health EDRM.

Theme	Findings from this study (Iran)	Findings from other studies	Key contributions of this study
Inadequate RP	Limited due to aging, low education, poverty, inequality, and linguistic diversity. Poor RC exacerbates issues. Focus on survival over preparedness.	Similar barriers noted by Clark PR et al. (2017): insufficient information, inadequate resources, and poor implementation of RP. Baytiyeh and Naja (2014) emphasize cultural and cognitive factors shaping RP.	Uniquely identifies the compounded effect of cultural and linguistic diversity on RP. Highlights rural–urban disparities in access to RC tools.
Community traumatization	Traumatization from past disasters reduces trust, fosters fatalistic attitudes, and shifts focus to recovery over preparedness. Structural factors like poor education in problem-solving intensify fatalism.	Baytiyeh and Naja (2014): Fatalistic beliefs hinder proactive actions. Similar psychological barriers reported in global studies.	Explores the structural causes of fatalism in communities, such as theological interpretations. Provides actionable insights into reducing trauma-related barriers.
Poor social capital	Weak networks, lack of trust, and top-down governance limit effective CP. Healthcare managers’ lack of transparency further weakens trust.	Yila et al. (2014): Top-down approaches disrupt social networks. Rivera and Nickels (2014): Contextual factors influence social capital.	Delivers a culturally specific analysis of how hierarchical structures and unmet promises erode social capital. Highlights the role of healthcare management transparency.

### 4.1 Inadequate RP

In Iran, many communities face significant challenges due to inadequate RP, which hinders effective CP in health EDRM. RP encompasses the ways in which individuals understand and evaluate potential disaster threats. When community members lack awareness of the risks present, they are less likely to engage in preventive measures to safeguard themselves and their communities. The study identifies various contextual factors that contribute to this issue. Our findings suggest that inadequate RP arises from individual attributes, such as aging and low education levels, as well as social factors like poverty and inequality. These elements collectively diminish community RP by shifting the focus from long-term preparedness to immediate survival needs. Additionally, older adults often exhibit lower RP due to entrenched beliefs and limited access to relevant information. Similarly, low educational attainment can restrict access to reliable risk information, thereby limiting disaster preparedness efforts. Furthermore, language and dialect differences can create inconsistent messaging and complicate RC, leading to confusion and mistrust in official guidelines. This phenomenon reflects challenges observed in culturally diverse communities, where linguistic diversity often exacerbates misunderstandings in disaster RP. Consistent with our findings, many studies have emphasized that cultural, social, cognitive, and political factors shape RP, potentially reducing a community’s ability to take preventive action (30–32). The findings of this study underscore a critical gap in community-level RA capabilities, which hinders both RP and CP. RA deficiencies—particularly in the form of limited specialized knowledge, unreliable information sources, and deficiency of essential tools and experience—significantly reduce communities’ resilience in the face of emergencies and disasters. This impact is especially severe in rural or less urbanized areas where access to formal education and disaster preparedness programs is restricted, exacerbating the existing challenges faced by these communities. The study by Clark et al. examines community perceptions of RA and the challenges in its implementation. This research highlights that communities’ understanding of risk and preparedness is often limited by insufficient information, inadequate resources, and implementation barriers, findings that align with our study. Additionally, the study also emphasizes the importance of resource allocation, education, and infrastructure development to improve RA in communities, enabling them to be better prepared for potential hazards (33). Furthermore, poor RC can shape inadequate RP. When communities receive information from fragmented sources with differing degrees of credibility, it can be confusing. The channels used for RC are not always accessible to everyone in the community. For example, in rural areas, limited access to digital communication tools and the internet can hinder the spread of critical risk information. Additionally, the information may not be conveyed in a way that is easily understandable or actionable for the community members, reducing its effectiveness. As other studies have demonstrated, this gap in effective RC can lead to several negative consequences (34, 35). Therefore, the ramifications of inadequate RP are significant. When communities fail to fully understand the potential health risks posed by disasters, they are less inclined to participate in critical preparedness activities, such as attending training sessions, participating in drills, or contributing to develop local emergency plans. This lack of participation can significantly weaken the effectiveness of health EDRM strategies, ultimately resulting in increased vulnerability and more severe health impacts when disasters occur.

### 4.2 Community traumatization

Community traumatization poses a significant challenge to effective CP in health EDRM within communities. Traumatization from previous disasters can deeply impact a community’s collective psyche, resulting in various psychological and social issues that impede proactive disaster preparedness efforts. Following traumatic events like earthquakes or floods, communities often experience widespread psychological distress, including feelings of hopelessness. This ongoing sense of vulnerability and fear can reduce the community’s willingness and capacity to engage in future DRM initiatives. Additionally, traumatization can shift the focus toward immediate needs and recovery, diverting attention from long-term preparedness efforts. In contemporary communities, multiple factors contribute to this traumatization. Increased feeling of unsafety in community, inadequate psychological preparedness, and reliance on a fatalistic attitude can all diminish the urgency regarding potential health risks. Community members may be wary of the government’s current preparedness initiatives if previous experiences featured poor responses or perceived failures. This wariness can hinder effective communication and collaboration between community members and disaster management authorities. Our study also revealed that fatalistic thinking is a significant barrier to proactive disaster preparedness and community resilience. Fatalism, the belief that human behavior is predetermined by external factors beyond one’s control, leads to a passive attitude and prevents meaningful contributions to preparedness efforts (36, 37). Stated differently, it results in community traumatization. The lack of effective health-related social structures, deficiency of problem-solving skills education, different interpretations of religious teachings, and belief in the inability to change conditions are influential structural factors in this area within our community. For instance, various theological interpretations contribute to a fatalistic worldview in many communities, where disasters are perceived as inevitable acts of fate or divine will. This mentality can deter people from taking preventive action because they believe nothing can be done to stop these events. As a result, community members may be less inclined to participate in health risk management initiatives, viewing them as futile. Numerous studies have demonstrated that fatalistic beliefs about the harm caused by natural disasters can deter individuals from taking the appropriate precautions against disasters (38–40). Earlier research by Baytiyeh and Naja, who claim that fatalistic attitudes may result in people’s mental capacities being reduced and communities being destroyed, support this finding (41).

### 4.3 Poor social capital

Poor social capital is a critical challenge that hinders CP in health EDRM. Social capital, encompassing networks, norms, and social trust that enable coordination and cooperation for mutual benefit, is crucial for effective community-based DRM. In communities with low social capital, trust and cohesion among members are often lacking (42). Our findings showed this deficiency can manifest in several ways, including weak community networks, weakened social norms, and declining trust within the community. When social capital is poor, it becomes difficult to mobilize community resources and foster the collective action needed for effective health risk management. Low social capital can also impede the development of trust between the community and external organizations involved in disaster management. Contextual factors such as top-down structures, a lack of transparency among healthcare managers, poor foresight of healthcare managers, and insufficient attention to the local community’s needs can weaken trust within the community. In many contexts, disaster management and health risk policies are formulated and implemented through hierarchical, top-down approaches, where higher authorities make decisions without adequate input from the local communities they aim to protect. This structure limits opportunities for community members to voice concerns, share knowledge, and contribute to risk management strategies. As noted by Yila et al., such top-down structures have been extremely problematic in numerous disastrous occurrences, disrupting existing communities and social networks (43). In addition, when there is a history of unfulfilled promises or ineffective interventions, community members may become skeptical of new initiatives, reducing their willingness to participate. These factors can undermine the effectiveness of community-based initiatives and hinder the active participation of community members. Social capital is highly dependent on resources and context, as explained by Rivera and Nickels, meaning that people’s networking capacities and purposes for building social capital vary in different contexts (44). Cheshire further notes that hostile relationships with proximate individuals can prevent networking in times of need. These findings align with our investigation’s outcomes (45).

## 5 Strengths and limitations of the study

This study is one of the few research efforts that qualitatively explores the challenges of CP in health EDRM from the participants’ own viewpoints. This approach offers valuable insights for planners and policymakers aiming to facilitate CP. Another strength of the study lies in the diverse participation of individuals, including healthcare managers, academics, and laypeople, which allowed for a multifaceted examination of the CP phenomenon. Although this is a qualitative study rooted in the experiences of Iranian participants, its findings can be applied to similar cultural contexts because emphasizing its trustworthiness, which further enhances its strength. This study faced several limitations. One key limitation was the difficulty in reaching national-level experts to take part in the research. In this instance, attempts were made to include individuals from middle management within health administration. Another limitation emerged during the interview phase; since some participants held high-demand management roles, accessing them was often challenging, and as a result, coordinating some of the interviews took longer than expected.

### 5.1 Implications to practice

The findings of this study can help policymakers develop clear guidelines to facilitate CP provision in health EDRM. Researchers can also use these results to provide more comprehensive evidence by conducting further studies on each of the identified barriers to CP.

## 6 Conclusion

The challenges identified in this study, namely inadequate RP, community traumatization, and poor social capital, underscore the multifaceted nature of CP in health EDRM. These findings highlight critical intervention areas and emphasize the necessity of an inclusive approach that values local knowledge, cultural contexts, and lived experiences. Although this study is rooted in the Iranian context, the barriers identified are relevant to other LMICs and diverse communities facing similar socio-economic and cultural challenges. To address these challenges, it is crucial for policymakers, healthcare managers, and community leaders to collaborate on strategies that can be adapted across different geographical and cultural contexts. For instance:

Educational campaigns and community workshops tailored to local contexts can enhance public awareness of disaster risks and promote proactive engagement.

Mental health services addressing post-disaster trauma can mitigate its impact on CP, a challenge prevalent in many disaster-prone regions worldwide.

Strengthening social capital through the establishment of local volunteer programs and neighborhood associations can foster trust, cohesion, and resilience.

The insights from this study can serve as a model for other countries, particularly those with cultural and social similarities, to improve disaster preparedness and response systems. Additionally, they can inform global disaster risk reduction strategies by offering an in-depth understanding of the interplay between socio-economic, cultural, and structural factors. Future research should focus on testing these strategies in varied contexts to create scalable, adaptable frameworks for enhancing CP in health EDRM globally. By addressing these key barriers, we can empower communities worldwide to actively safeguard their health and well-being in the face of disasters.

## Data Availability

The original contributions presented in the study are included in the article/supplementary material, further inquiries can be directed to the corresponding author.
